# Diffuse large B-cell lymphoma presenting as acute adrenal hemorrhage

**DOI:** 10.1007/s44313-025-00064-8

**Published:** 2025-03-19

**Authors:** Fabio Torres, Uriel Suárez, Paola Pizano

**Affiliations:** 1 Department of Hematology, Fundación Jiménez Díaz University Hospital (FJD, UAM), Universidad Autónoma de Madrid, Madrid, Spain; 2 Department of Radiology, Fundación Jiménez Díaz University Hospital (FJD, UAM), Universidad Autónoma de Madrid, Madrid, Spain; 3 Av. de los Reyes Católicos, 2, Moncloa-Aravaca, 28040 Madrid, Spain

**Keywords:** Abdomen, Acute, Anemia, Adrenal gland diseases, Lymphoma

**Figure:**
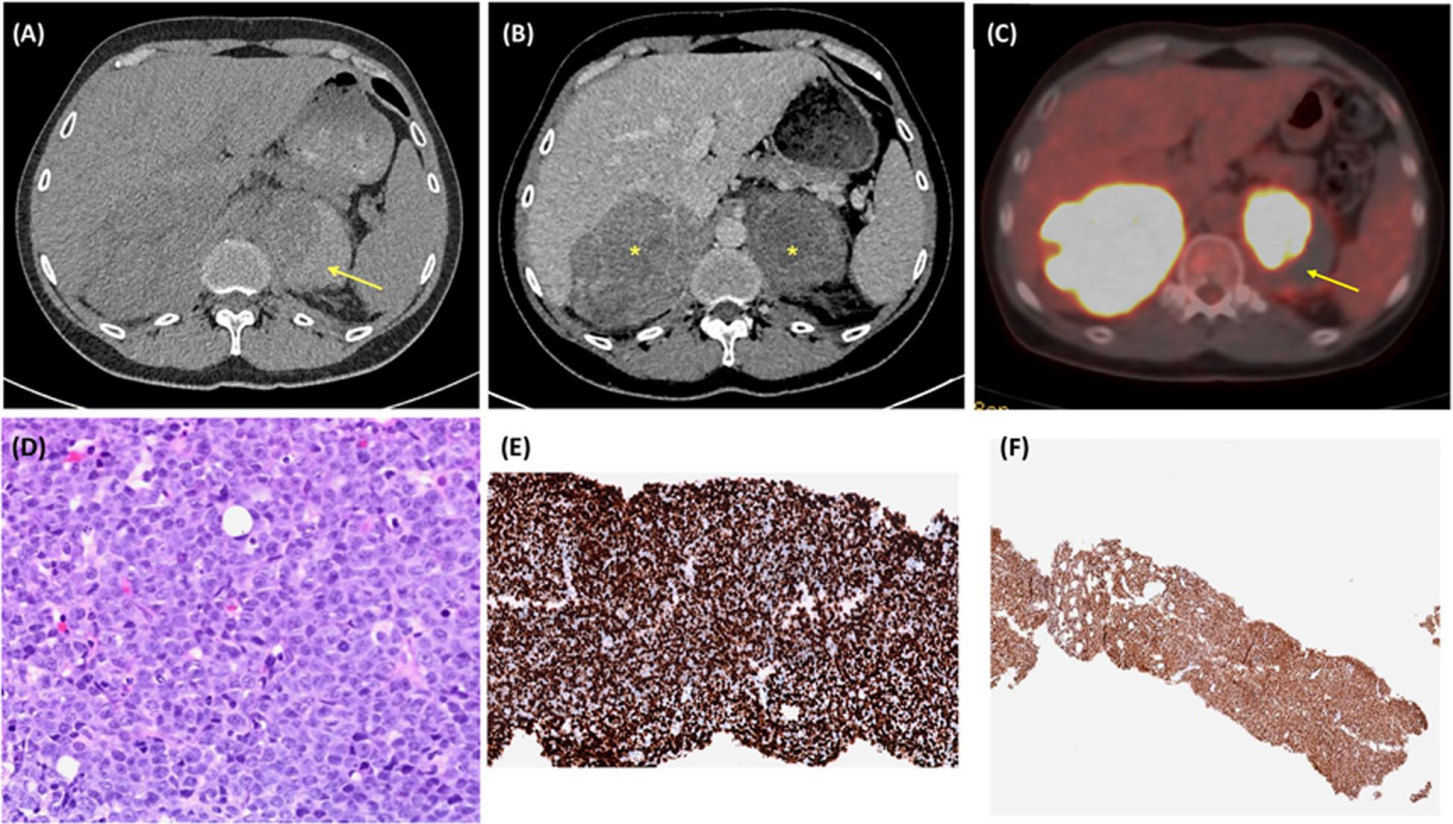
Figure 1. (A) Transverse non-contrast abdominal computed tomography (CT) with a hyperdense content within the left adrenal gland (yellow arrow) secondary to adrenal bleeding. (B) Transverse contrast-enhanced abdominal CT with a massive enlargement of adrenal glands (asterisks). (C) Positron emission tomography-CT with increased fluorodeoxyglucose (FDG) uptake by adrenal glands suggestive of lymphoma (yellow arrow shows a zone with decreased FDG uptake due to acute adrenal bleeding). (D) Hematoxylin and eosin staining of right adrenal biopsy. This section did not include zones with active adrenal hemorrhage (hematoxylin–eosin staining × 20). (E–F) MYC (× 10) and MUM1 (× 2) immunohistochemistry

## Case presentation

A 45-year-old man presented to the emergency department with a 1-day history of abdominal pain in the left flank and vomiting. No hypotension, rebound tenderness, or hepatosplenomegaly was observed. Laboratory studies revealed elevated levels of lactate dehydrogenase (LDH; 523 IU/L) and low hemoglobin (11·9 g/dL). Abdominal computed tomography (CT) revealed bulky adrenal masses with left adrenal hemorrhage (AH; Fig. 1A and B). No lymphadenopathy was observed. Positron emission tomography-CT revealed avid fluorodeoxyglucose (FDG) adrenal lesions and a zone with decreased FDG uptake due to acute AH (Fig. 1C). An adrenal biopsy revealed diffuse growth of large-sized atypical lymphoid cells with irregular nuclei, vesicular chromatin, and scanty cytoplasm (Fig. 1D). The immunostaining was positive for CD20 and BCL2. MYC and MUM expression were observed in approximately 100% of the cells (Fig. 1E and F) without CD10. The final diagnosis was diffuse large B-cell lymphoma, subtype activated B-cell-like. The abdominal pain improved with rituximab, cyclophosphamide, hydroxydaunorubicin, oncovin, and prednisone treatment.

Lymphoma is a differential diagnosis of acute abdominal pain in patients with elevated LDH levels and adrenal bleeding. In most patients with AH, the diagnosis is made incidentally using imaging tests [1, 2]. The clinical presentation ranges from nonspecific abdominal pain to adrenal insufficiency. Lymphomas are an emerging cause of this complication. Secondary adrenal lymphoma involvement is the most common etiology, followed by primary adrenal lymphoma [3]. This diagnosis should be considered in all patients with adrenal hemorrhage, even in the absence of adenopathy or splenomegaly.

## Data Availability

No datasets were generated or analysed during the current study.
